# The EEF1A2 gene expression as risk predictor in localized prostate cancer

**DOI:** 10.1186/s12894-017-0278-3

**Published:** 2017-09-18

**Authors:** Thomas Stefan Worst, Frank Waldbillig, Abdallah Abdelhadi, Cleo-Aron Weis, Maria Gottschalt, Annette Steidler, Jost von Hardenberg, Maurice Stephan Michel, Philipp Erben

**Affiliations:** 1 Department of Urology, University Medical Centre Mannheim, University of Heidelberg, Theodor-Kutzer-Ufer 1-3, 68167 Mannheim, Germany; 2 Institute of Pathology, University Medical Centre Mannheim, University of Heidelberg, Theodor-Kutzer-Ufer 1-3, 68167 Mannheim, Germany

**Keywords:** EEF1A2, Prostate cancer, Risk stratification, Biomarker, Expression, Outcome prediction

## Abstract

**Background:**

Besides clinical stage and Gleason score, risk-stratification of prostate cancer in the pretherapeutic setting mainly relies on the serum PSA level. Yet, this is associated with many uncertainties. With regard to therapy decision-making, additional markers are needed to allow an exact risk prediction. *Eukaryotic translation elongation factor 1 alpha 2* (EEF1A2) was previously suggested as driver of tumor progression and potential biomarker. In the present study its functional and prognostic relevance in prostate cancer was investigated.

**Methods:**

EEF1A2 expression was analyzed in two cohorts of patients (*n* = 40 and *n* = 59) with localized PCa. Additionally data from two large expression dataset (MSKCC, Cell, 2010 with *n* = 131 localized, *n* = 19 metastatic PCa and TCGA provisional data, *n* = 499) of PCa patients were reanalyzed. The expression of EEF1A2 was correlated with histopathology features and biochemical recurrence (BCR). To evaluate the influence of EEF1A2 on proliferation and migration of metastatic PC3 cells, siRNA interference was used. Statistical significance was tested with t-test, Mann-Whitney-test, Pearson correlation and log-rank test.

**Results:**

qRT-PCR revealed EEF1A2 to be significantly overexpressed in PCa tissue, with an increase according to tumor stage in one cohort (*p* = 0.0443). In silico analyses in the MSKCC cohort confirmed the overexpression of EEF1A2 in localized PCa with high Gleason score (*p* = 0.0142) and in metastatic lesions (*p* = 0.0038). Patients with EEF1A2 overexpression had a significantly shorter BCR-free survival (*p* = 0.0028). EEF1A2 expression was not correlated with serum PSA levels. Similar results were seen in the TCGA cohort, where EEF1A2 overexpression only occurred in tumors with Gleason 7 or higher. Patients with elevated EEF1A2 expression had a significantly shorter BCR-free survival (*p* = 0.043). EEF1A2 knockdown significantly impaired the migration, but not the proliferation of metastatic PC3 cells.

**Conclusion:**

The overexpression of EEF1A2 is a frequent event in localized PCa and is associated with histopathology features and a shorter biochemical recurrence-free survival. Due to its independence from serum PSA levels, EEF1A2 could serve as valuable biomarker in risk-stratification of localized PCa.

## Background

In industrialized countries prostate cancer (PCa) is the cancer entity with the highest incidence in men. Though most tumors can be cured in early stages or are insignificant, without need for any treatment at all, around the world over 250,000 patients die from PCa per year [1]. Due to an increasing awareness, PCa screening has become more frequent throughout the last decades. Besides digital rectal exam, prostate specific antigen (PSA) is the current number one screening tool for PCa. But its value is debated controversially [2], due to limited PCa specificity and imprecise prediction of PCa aggressiveness [3]. Mixed models implementing PSA level, biopsy Gleason grade and clinical stage are typically utilized to estimate the individual risk for aggressive PCa with rapid progression along with early metastasization and – together with patient age and risk factors – lead to therapy decision in order to avoid overtreatment by treating only relevant carcinomas [4].

Since these estimations are still imprecise, further markers, either blood- or urine-based or derived from biopsy tissue samples, are needed to specify the individual patient’s risk and to facilitate therapy decision-making. Yet, PCa markers intensively studied during the last decade (e.g. genetic markers like the fusion gene TMPRSS2:ERG, circulating tumor cells or urine PCA3 test) are not used in clinical routine [3, 5, 6].

EEF1A2 (*eukaryotic translation elongation factor 1 alpha 2*) is part of a complex that enzymatically delivers aminoacyl tRNAs to the ribosome and is mainly expressed in brain, heart and skeletal muscles [7]. In general it is reported to favor oncogenesis by stimulating the phospholipid signaling and the Akt-dependent cell migration [8]. Besides its role in cancer, EEF1A2 mutations are associated with characteristic facial features, intellectual disability, autistic behavior and epilepsy [9].

There is also evidence, that EEF1A2 expression is predictive for patient outcome in various epithelial cancer entities [10–12]. In PCa one study found the more ubiquitously expressed isoform EEF1A1 to be overexpressed in peri-metastatic osteoblasts in PCa bone metastasis, compared to normal osteoblasts [13]. Another study found an overexpression of EEF1A2 in PCa tissue compared to matched benign tissue in a small preliminary cohort [14]. In the same study the authors could show an overexpression of EEF1A2 to inhibit apoptosis in metastatic PCa cells. Therefore they claimed EEF1A2 to be a hallmark for PCa progression. The impact of EEF1A2 expression – both on the mRNA and on the protein level – on clinical outcome has not been investigated, yet.

To validate recent findings about EEF1A2 overexpression in PCa on the mRNA level, sensitive qRT-PCR techniques were used on two independent cohorts of patients with localized PCa. Subsequent in silico analysis of RNA expression datasets served for validation. To gain further insight into the biological function of EEF1A2 in PCa siRNA interference experiments were conducted in vitro.

## Methods

### Cohorts and patient samples

qRT-PCR was used to asses EEF1A2 RNA expression in a cDNA array (Origene, Rockville, MD, USA; *n* = 40 PCa patients and *n* = 8 benign control samples). Patient characteristics of this cohort are shown in Table 1.

**Table 1:** Patient characteristics of the cDNA Array purchased from Origene

Parameter	*n*
Patients with PCa	40 (mean age 62.8 ± 8.2)
T stage	
T1	–
T2	22
T3	12
T4	–
n/a	6
N stage	
N0	20
N1	2
Nx	18
Gleason score	
5	2
6	8
7a	14
7b	8
8	3
9	4
n.a.	1
Control patients	8 (64.0 ± 10.9)

To further correlate EEF1A2 expression with clinical follow up data, a cohort of 59 patients who underwent radical prostatectomy in the Department of Urology of the Mannheim Medical Center between 1998 and 2001 was analyzed. Patient data of this cohort is given in Table 2. Prostate tissue specimens from patients who underwent cystoprostatecomy or transurethral resection of the prostate, with histologically proven tumor-free prostate, served as controls. All experiments conducted in this retrospective analysis were in accordance with the institutional ethics review board (ethics approval 2013-845R-MA).

**Table 2:** Patient characteristics of the cohort recruited in Mannheim

Parameter	*n*
Patients with PCa	59 (mean age 62.9 ± 6.9)
T stage	
T1	–
T2	23
T3	33
T4	3
N stage	
N1	5
N0	47
Nx	7
Gleason score	
3	1
4	0
5	10
6	15
7a	15
7b	4
8	5
9	3
10	2
n.a. due to prior antihormonal therapy	4
average serum PSA level	13.3 ng/ml (2.8–73.0 ng/ml)
Control patients	15 (mean age 67.2 ± 11.3)

### RNA-extraction, cDNA-synthesis and qRT-PCR from patient samples

Sections of tumor-bearing or tumor-free FFPE prostate tissue specimens were stained with hematoxylin and eosin and reviewed by a trained pathologist. Areas with at least 70% of tumor or tumor-free areas from control patients were marked and macrodissected from subsequent unstained 10 μm sections. RNA was extracted using the XTRAKT FFPE kit (Stratifyer, Cologne, Germany), as recommended by the manufacturer. In brief 150 μl of lysis buffer were added to the tissue sample and incubated for 30 min at 80 °C while shaking. After cooling down to 65 °C 50 μl of proteinase K (Roche) were added and incubated for 30 min at 65 °C while shaking. Subsequently 800 μl of MagiX-RNA buffer and 40 μl of MagiX-RNA beads were added and incubated at room temperature for 15 min while shaking. The mixed samples were put on a magnetic rack and washed three times. Finally the RNA was eluted in 100 μl of elution buffer. RNA samples were stored at −80 °C.

To receive a greater yield of target specific transcripts and to reduce contamination with other amplified cDNA sequences, we used a multiplexed specific cDNA synthesis with equimolar pooling of transcript specific reverse PCR primers (primer sequences see below). Superscript III (Life technologies) was used as reverse transcriptase at 55 °C for 120 min, followed by an incubation at 70 °C for 15 min. cDNA was immediately used for qRT-PCR or stored at −20 °C.

In the cDNA array the expression of EEF1A2 was determined in relation to the housekeeping gene Calmodulin 2 (CALM2). Intron spanning primer pairs (CALM2: forward GAGCGAGCTGAGTGGTTGTG reverse AGTCAGTTGGTCAGCCATGCT amplicon length 72 nt; EEF1A2: forward GGACCATTGAGAAGTTCGAGA, reverse AGCACCCAGGCATACTTGAA, amplicon length 70 nt) compatible with the Universal Probe library (Roche Diagnostics) were designed using the primer3 algorithm [15]. In brief 10 μl of TaqMan Fast Universal PCR Mastermix (Life technologies), 0.75 μl of forward and reverse primer each (300 nM) (MWG Eurofins, Ebersberg, Germany) 0.5 μl of PCR probe (200 nM) (Roche Diagnostics) and 6 μl of nuclease free H_2_O were added to 2 μl of cDNA template each. Subsequently 40 cycles of amplification with 1 s of 95 °C and 20 s of 60 °C were conducted on a Step One Plus qRT-PCR cycler (Applied Biosystems, Waltham, MA, USA). To allow a higher input of cDNA, the volume of primers was halved for qRT-PCR analysis of the Mannheim cohort. RNA-expression was calculated with the 2^(−ΔΔcT)^-method [16].

### Datamining and in silico validation

From the online platform CBioPortal [17] RNA expression data (z-score normalized) of two datasets also comprising clinical follow-up were downloaded: The MSKCC dataset consists of 131 primary an 19 metastatic tumor samples *(Taylor* et al.*, Cancer Cell, 2010)* [18]. The TCGA (The Cancer Genome Atlas) dataset includes expression data of 499 primary PCa samples. EEF1A2 RNA expression was stratified by tumor characteristics and correlated with BCR-free (biochemical recurrence) survival and serum PSA levels.

### Cell culture, siRNA knockdown and knockdown validation

Human PC3 metastatic PCa cells were obtained from ATCC (Wesel, Germany) and grown under standard conditions in DMEM (Life Technologies, Carlsbad, CA, USA) supplemented with 10% FCS (Sigma Aldrich, St. Louis, LA, USA). siGENOME pooled and individual siRNAs against EEF1A2 (No 1 GTACAAGATTGGCGGCATT, No 2 TCAAGAAGATCGGCTACAA, No 3 CTACAAATGCGGAGGTATT, No 4 ATGCGGAGGTATTGACAAA) were transfected using Dharmafect I transfection reagent (Dharmacon, Lafayette, CO, USA). Dharmacon non-targeting siRNA were used as negative control. Briefly cells were detached, harvested, spun down and diluted to the desired concentration. Meanwhile siRNAs were diluted to a target concentration of 30nMol in pure RPMI (Life Technologies) and incubated for 10 min at room temperature. Dharmafect I was diluted 1:1000 in RPMI. After 10 min diluted siRNA and transfection reagent were mixed 1:1 and again incubated at room temperature for 30 min. Hereafter cell suspension was added to the transfection mix 3:1 and incubated at 37 °C.

qRT-PCR was conducted to validate knockdown of EEF1A2. RNA-extraction was performed using the RNeasy Mini Kit (Qiagen, Hilden, Germany) as recommended by the manufacturer. cDNA-Synthesis was performed as described previously [19]: in brief 40 μl of diluted RNA were mixed with 4 μl of 5 mg/ml pdN6 random primers, 4 μl of 10 mM dNTP Mix, 16 μl of 5× M-MLV buffer, 8 μl of 0.1 M RNase inhibitor, 4 μl of 0.1 M DTT and 4 μl of M-MLV reverse transcriptase (all from Roche Diagnostics, Basel, Switzerland). After an incubation for 2 h at 37 °C and a deactivation step of 5 min at 65 °C, cDNA was directly used for qRT-PCR or stored at −20 °C. qRT-PCR analyses were performed using the same primers, reagents and PCR protocol as described for tissue sample analyses.

### Proliferation assay

PC3 cells were seeded and transfected following the protocol described above in 96-well plates (4500 cells in 100 μl/well). After 24 h the supernatant was replaced by 100 μl of fresh growth medium (DMEM with 10% FCS). After further 24, 48 and 72 h of incubation 10 μl of MTT-reagent (Promega, Mannheim, Germany) were added to each well and incubated for 3 h at 37 °C. Absorption measurement at 570 nm was done with an Infinite M1000 Pro plate reader (Tecan, Männerdorf, Switzerland).

### Scratch assay

Using the same transfection protocol, PC3 cells were seeded in 24-well plates (250,000 cells in 1 ml of DMEM with 10% FCS per well). The medium was changed 24 h after transfection. Again 24 h later a defined scratch was introduced in the center of the well with a sterile 200 μl pipette tip and the medium was changed again. The scratch was photographed at 10× magnification. Subsequent images were acquired after further 24, 48 and 72 h. The cell free space in the scratch area was calculated with the open source software *tscratch* (ETH Zürich, Switzerland) [20]. The free area 24, 48 and 72 h after scratch were normalized to the initial scratch size.

### Statistics

Statistical calculations were performed using Prism 6 (Graphpad, La Jolla, USA). Mann-Whitney-Test was used for calculation of inter-group expression changes in patient cohorts analyzed with qRT-PCR and in silico data. Outcome correlations were done using the log-rank test. Correlations with the PSA serum level were performed using Pearson correlation. Parametric t-test was used for in vitro assays. *P*-values ≤0.05 were deemed significant.

## Results

### qRT-PCR analysis indicates EEF1A2 overexpression in PCa patients

**Fig. 1:**
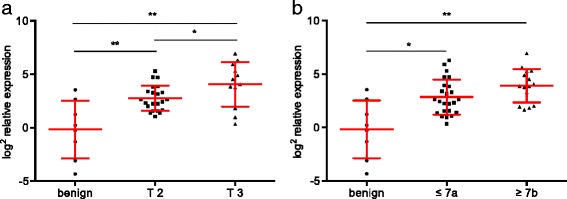
qRT-PCR analysis in a panel of 40 PCa patients showed an overexpression of EEF1A2 in localized tumor samples. **a**) EEF1A2 expression was significantly dependent of tumor stage. **b**) The averages expression was higher in tumors with a Gleason score ≥ 7b, but this difference did not reach significance

To investigate the expression of EEF1A2 in PCa qRT-PCR expression analyses in a cDNA array of 40 patients with localized PCa were performed. Compared to benign tissue samples EEF1A2 was overexpressed 6.76-fold in T2 tumors (*p* = 0.006) and 16.6-fold in T3 tumors (*p* = 0.0011) (Fig. 1a). The expression was also significantly higher in T3 compared to T2 tumors (*p* = 0.0443). Similar results were seen after stratification for Gleason grade (Fig. 1b). Tumors with a Gleason score ≤ 7a had a 7.22-fold higher expression of EEF1A2 (*p* = 0.0064). In tumors with Gleason ≥7b a 15.09-fold higher expression was seen (*p* = 0.0004). Though EEF1A2 expression was higher in tumors with higher Gleason score, no significant difference was seen between tumors with Gleason score ≤ 7a and ≥7b (*p* = 0.0541).

**Fig. 2:**
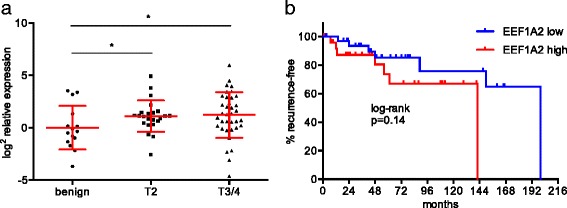
**a**) In a cohort of 59 patients, undergoing radical prostatectomy EEF1A2 was overexpressed in PCa. There was no significant difference between tumor stages. **b**) Patients with high EEF1A2 expression in the primary tumor in tendency showed a shorter recurrence-free survival. Yet, log-rank test showed this difference not to be significant

Since the analyzed cDNA array did not provide clinical follow-up data, the expression of EEF1A2 was further evaluated in a cohort of 59 patients treated with radical prostatectomy in the Mannheim Medical Center. EEF1A2 was significantly overexpressed in these tumor samples (Fig. 2a). In T2 tumors EEF1A2 was 2.16-fold overexpressed compared to benign controls (*p* = 0.0277). In T3/4 tumors a 2.23-fold overexpression was observed (*p* = 0.0325). In this dataset no significant difference in expression was seen between T2 tumors and locally advanced tumors.

EEF1A2 expression did not correlate with the serum PSA level (*r* = −0.02058; *p* = 0.8771). Kaplan-Meier analysis revealed a slightly shorter recurrence-free survival of patients with high EEF1A2 expression (mean follow-up 67.5 months, ± 51.2 months, Fig. 2b). Yet, log-rank test showed this difference not to be significant (*p* = 0.14). Taken together, these results point to a potential relevance of EEF1A2 risk predictor in localized PCa.

### In silico analyses reveal EEF1A2 as outcome predictor in localized PCa

**Fig. 3:**
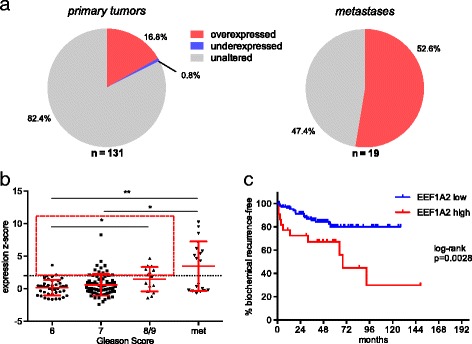
In silico *validation of EEF1A2* **a**) Reanalysis of the microarray dataset by *Taylor* et al. revealed EEF1A2 to be overexpressed in 16.9% of localized PCA and in 52.6% of metastatic PCa (dashed line indicates an expression z-score of 2 compared to benign tissue). **b**) EEF1A2 overexpression was grade dependent in localized PCa and highest in metastases. **c**) Localized tumors with high EEF1A2 expression (indicated by a red square in **b**) had a significantly shorter biochemical recurrence-free survival

To validate the expression of EEF1A2 in large cohorts of PCa patients, in silico analyses on the RNA expression microarray dataset by *Taylor* et al. *and on the RNA sequencing data of the TCGA cohort* were conducted*.* In the *Taylor* et al. dataset 16.8% of patients with localized PCa had a z-score ≥ 2, compared to benign controls (Fig. 3a). In metastatic tumor samples EEF1A2 overexpression was found in 52.6% of the patients. The highest expression was seen in PCa metastases (*p* = 0.0038 when compared with Gleason 6 localized tumors; Fig. 3b). Focusing on primary tumors, overexpression of EEF1A2 was a rare event in Gleason 6 tumors (2/41, 4.9%), and rather seldom in Gleason 7 tumors (13/74, 17.6%). Among Gleason 8/9 tumors, 7 out of 15 (46.7%) had an EEF1A2 z-score > 2. In line with the qRT-PCR data presented here, this indicated a higher expression of EEF1A2 in more aggressive tumors (*p* = 0.0142 for Gleason 8/9 tumors vs. Gleason 6 tumors). Pearson correlation of EEF1A2 with the serum PSA levels at the time of surgery again showed no correlation (*r* = 0.1590; *p* = 0.0708).

Then EEF1A2 expression in primary tumors was correlated with patient outcome. Interestingly patients with an expression z-score of EEF1A2 ≥ 2 had a significantly shortened BCR-free survival compared to patients without EEF1A2 overexpression (*p* = 0.0028, mean follow-up 48.5 months, ± 29.6 months, Fig. 3c).

**Fig. 4:**
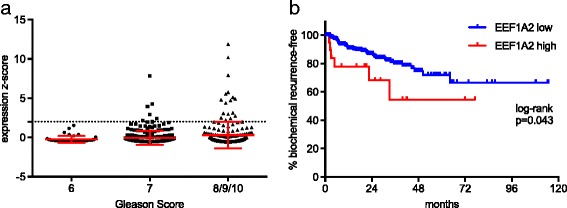
**a**) EEF1A2 expression in the TCGA cohort (*n* = 499; dashed line indicated an expression z-score of 2). **b**) Tumors with an elevated EEF1A2 expression had a significantly shorter BCR-free survival

In the TCGA cohort (*n* = 499) there was no significant difference in the average expression of EEF1A2 according to the Gleason Score (Fig. 4a). Yet, only tumors with a Gleason Score of 7 or higher showed an overexpression of EEF1A2 (z-score ≥ 2). Tumors with an elevated EEF1A2 expression had a significantly shorter BCR-free survival (*p* = 0.043; Fig. 4b). The serum PSA levels of the patients in this cohort are not available and could therefore not be correlated with the EEF1A expression.

### EEF1A2 is functionally involved in PCa migration but not in proliferation

**Fig. 5:**
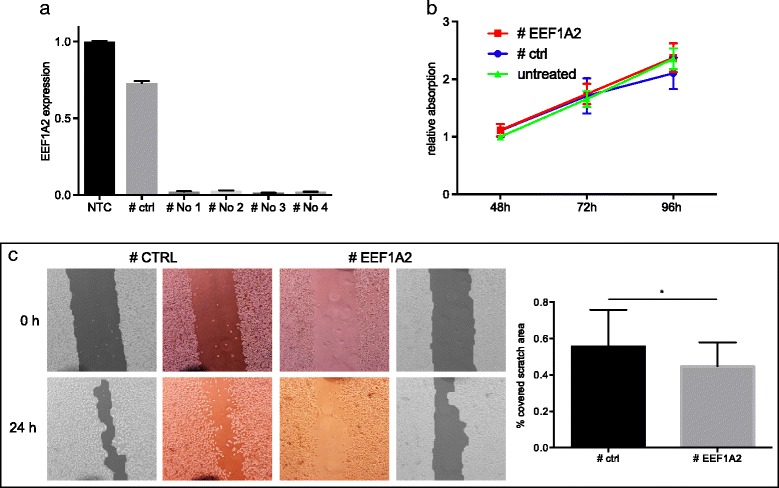
In vitro testing of EEF1A2. **a**) qRT-PCR validation of siRNA-mediated knockdown of EEF1A2. **b**) EEF1A2 knockdown did not alter tumor cell proliferation in PC3 cells, **c**) but significantly hampered PC3 cell migration in a scratch wound healing assay

To get a first insight into the function of EEF1A2 in PCa, transient transfection experiments were conducted in metastatic PC3 cells. qRT-PCR verified a strong knockdown effect for all used siRNAs (Fig. 5a). For further experiments siRNA No 3, which produced the strongest knockdown, was used.

In the MTT assay knockdown of EEF1A did not lead to a significant alteration of PC3 cell proliferation (Fig. 5b). By using scratch wound healing assay the influence of EEF1A2 knockdown on PC3 cell migration was studied. A significant reduction of migration in cells with EEF1A2 knockdown was observed (*p* = 0.035). After 24 h, 55.6% of the initial scratch area were covered by control-transfected cells, whereas only 44.7% were covered in EEF1A2-knockdown cells (Fig. 5c).

## Discussion

The study aimed to determine the EEF1A2 expression in PCa tissue, to test for its potential relevance as risk predictor in localized PCa and to gain insight into its functional role of in PCa.

EEF1A2 was overexpressed in localized PCa with a stage-dependent increase in one of the two cohorts tested with qRT-PCR. In silico analyses of one *Taylor* et al. dataset confirmed the overexpression of EEF1A2 in aggressive localized PCa. The expression was dependent of the Gleason Score and patients with a high expression in localized PCa had a significantly shorter BCR-free survival. In the TCGA dataset EEF1A2 overexpression only occurred in tumors with a Gleason Score of 7 or higher and again tumors with an elevated EEF1A2 expression had a significantly shorter BCR-free survival. qRT-PCR results also point to a potential association with recurrence-free survival. Though the cohorts analyzed with qRT-PCR were of limited patient number they are to date the largest series, in which EEF1A2 expression was profiled in PCa with a specific method. Furthermore this is the first study correlating clinical follow-up data with EEF1A2 expression in PCa.

Data on metastatic tumors has not been reported, yet. By reanalyzing existing microarray data, we revealed EEF1A2 to be overexpression in more than 50% of PCa metastases, underlining its association with an aggressive PCa phenotype.

Since EEF1A2 expression was not correlated with serum PSA levels it might provide additional value in PCa risk stratification of localized PCa as a tissue based marker, e.g. from prostate biopsy samples. In this setting qRT-PCR offers several advantages compared to conventional immunohistochemistry. qRT-PCR is more sensitive, allows a better quantification and therefore contributes to a better comparability between samples. Furthermore results are less observer-dependent. However, qRT-PCR does not give information about the expression pattern in the tissue.

The results of the present study are in accordance with results from the recent literature. *Scaggiante* et al. [21] deemed EEF1A2 as a marker for prostate cell transformation and a potential hallmark of cancer progression, since they found it overexpressed in metastatic PCa cell lines, compared with benign prostate cells. Additionally they found it to be overexpressed both in PCa tissue and peritumoral stroma in a small series of nine PCa patients.

*Sun* et al. [14] found a significantly higher RNA expression of EEF1A2 in 26 out of 30 primary PCa samples compared to matched control samples. Using the same cohort they could validate these results on the protein level using immunohistochemistry. When they correlated their immunohistochemistry results with clinical features (age, PSA >/≤ 50 ng/ml, Gleason >/≤ 7, stages >/≤ T2), they did not find a significant correlation. Unfortunately they only performed these correlations on the protein level and did not further stratify their cohort. Additionally the number of patients in this study was comparably small. Therefore a correlation of EEF1A2 expression and the analyzed clinical features might have been missed. They also did not implement any follow-up data, making an outcome prediction impossible.

Reports on EEF1A2 expression in other tumor entities show differing results. Interestingly some studies attribute a higher expression of EEF1A2 with a poor prognosis, whilst others found it to be associated with a favorable outcome. In a large cohort of 438 primary breast cancer specimen, absence of EEF1A2 protein expression was a predictor of poor outcome [10]. EEF1A2 expression was not associated with other established prediction markers like HER-2 protein expression, tumor size, lymph node status, and estrogen receptor expression.

Controversially negative staining for EEF1A2 was a predictor for poor outcome in patients with non-small cell lung cancer [22]. In pancreatic ductal adenocarcinoma elevated expression of EEF1A2 was associated with nodal metastasis, perineural invasion and worse prognosis [11].

In vitro testing via siRNA-interference of EEF1A2 revealed a reduction of PC3 cell migration, indicating a potential tumor promoting function. Results on the impact of EEF1A2 on PCa cell migration have not been reported so far. The growth of PC3 cells was not altered. Interestingly another study showed a significant reduction of PCa cell growth and colony formation upon knockdown of EEF1A2 [14], which is partly controversial to the findings in the present study. Yet, differing results might be caused by different assay conditions like the growth media and siRNAs used.

A reduced growth of PCa cells upon EEF1A2 knockdown is in line with the proposed pro-oncogenic function of EEF1A2 in PCa. The same study also found an increase in apoptosis upon knockdown of EEF1A2, which had already been described in studies on other solid cancer entities [23, 24].

In pancreatic cancer EEF1A2 overexpression also resulted in an activation of AKT and led to an overexpression of the matrixmetallo-protease MMP9, which is a key player in extracellular matrix reorganization in context of cancer progression [12].

In hepatocellular carcinoma EEF1A2 was shown to inactivate P53 via an upstream activation of the PI3K/AKT/mTOR-pathway [25]. This gives rationale to a druggability of EEF1A2-dependent tumor growth in PCa with mTOR inhibitors, which are already in clinical use e.g. for metastatic renal cell carcinoma, breast cancer, neuroendocrine tumors and certain lymphomas [26, 27]. Actually, several early clinical trials using mTOR inhibitors in metastatic PCa are ongoing but merely show discouraging results [28–30]. A recent systematic review suggested reciprocal feedback mechanisms between PI3K and androgen receptor signaling to be causative for this and proposed a combinatorial targeted therapy of PI3K, mTOR and the androgen receptor [30].

## Conclusion

qRT-PCR and in silico expression analyses confirm recent reports from smaller series about EEF1A2 overexpression in localized PCa. Furthermore this is the first study describing EEF1A2 expression to be stage- and grade dependent and EEF1A2 overexpression to be predictive for the outcome of patients with localized PCa. Since EEF1A2 expression is not correlated with serum PSA levels, it might serve as an additional biomarker for PCa risk stratification. Further prospective studies, investigating EEF1A2 expression e.g. in needle biopsy samples are needed to evaluate its value as prognostic biomarker. In vitro experiments give an outlook on the functional role of EEF1A2 in PCa.

## Abbreviations

BCR: Biochemical recurrence
EEF1A2: Eukaryotic translation elongation factor 1 alpha 2
MSKCC: Memorial Sloan Kettering Cancer Center
PCa: Prostate cancer
qRT-PCR: Quantitative real-time PCR
TCGA: The Cancer Genome Atlas
TMPRSS2:ERG: Transmembrane protease, serine 2:ETS-related gene
